# Relationship between the respiratory microbiome and the severity of airflow limitation, history of exacerbations and circulating eosinophils in COPD patients

**DOI:** 10.1186/s12890-019-0867-x

**Published:** 2019-06-24

**Authors:** Laura Millares, Sergi Pascual, Concepción Montón, Marian García-Núñez, Cristina Lalmolda, Rosa Faner, Carme Casadevall, Laia Setó, Silvia Capilla, Amàlia Moreno, Ady Angélica Castro-Acosta, Carlos José Alvarez-Martinez, Oriol Sibila, Germán Peces-Barba, Borja G. Cosio, Alvar Agustí, Joaquim Gea, Eduard Monsó

**Affiliations:** 1 Fundació Parc Taulí- Institut d’ Investigació i Innovació Parc Taulí (I3PT), Barcelona, Spain; 2 Centro de Investigación Biomédica en Red de Enfermedades Respiratorias (CIBERES), Instituto de Salud Carlos III (ISCIII), Madrid, Spain; 3 Universitat Autònoma de Barcelona, Esfera UAB, Barcelona, Spain; 4 Respiratory Medicine Department, Hospital del Mar – IMIM, Barcelona, Spain; 5 Department of Respiratory Medicine, Hospital Universitari Parc Taulí, Parc Taulí 1, 08208, Sabadell, Barcelona, Spain; 6 Health Services Research on Chronic Diseases Network- REDISSEC, Galdakao, Spain; 7 Barcelona Respiratory Network, Barcelona, Spain; 8 DCEXS, Universitat Pompeu Fabra, Barcelona, Spain; 9 Department of Microbiology, Hospital Universitari Parc Taulí, Sabadell, Spain; 10 Department of Respiratory Medicine, Hospital Universitario 12 de Octubre, Madrid, Spain; 11 Respiratory Department, Biomedical Research Institute Sant Pau (IIB-Sant Pau), Hospital de la Santa Creu i Sant Pau, Barcelona, Spain; 12 Department of Respiratory Medicine, Fundación Jiménez Díaz, Madrid, Spain; 13 Department of Respiratory Medicine, Hospital Universitari Son Espases-IdISBa, Mallorca, Spain; 14 Institut Respiratori, Hospital Clinic, IDIBAPS, Universidad de Barcelona, Barcelona, Spain; 15 Department of Medicine, Universitat Autònoma de Barcelona (UAB), Barcelona, Spain

**Keywords:** Bacterial community, Diversity, Eosinophils, Exacerbations, Sputum, Stable COPD

## Abstract

**Background:**

The respiratory microbiome is altered in COPD patients but its relationship with core components of the disease, such as the severity of airflow limitation, the frequency of exacerbations or the circulating levels of eosinophils, is unclear.

**Methods:**

Cross-sectional study comprising 72 clinically stable COPD patients (mean age 68 [SD 7.9] years; FEV1 48.7 [SD 20.1]% of reference) who provided spontaneous sputum samples for 16S rRNA gene amplification and sequencing. The microbiome composition was analysed with QIIME.

**Results:**

We observed that: (1) more severe airflow limitation was associated with reduced relative abundance (RA) of *Treponema* and an increase in *Pseudomonas*; (2) patients with ≥2 exacerbations the previous year showed a significantly different bacterial community with respect to non-exacerbators (*p* = 0.014), with changes in 13 genera, including an increase of *Pseudomonas,* and finally, (3) peripheral eosinophils levels ≥2% were associated with more diverse microbiome [Chao1 224.51 (74.88) vs 277.39 (78.92) *p* = 0.006; Shannon 3.94 (1.05) vs 4.54 (1.06) *p* = 0.020], and a significant increase in the RAs of 20 genera.

**Conclusion:**

The respiratory microbiome in clinically stable COPD patients varies significantly according to the severity of airflow limitation, previous history of exacerbations and circulating eosinophils levels.

**Electronic supplementary material:**

The online version of this article (10.1186/s12890-019-0867-x) contains supplementary material, which is available to authorized users.

## Summary at a glance

Core components of COPD such as airflow limitation, history of previous exacerbations and level of circulating eosinophils have an impact in the bronchial respiratory microbiome of clinically stable COPD patients.

## Background

Chronic obstructive pulmonary disease (COPD) is a heterogeneous disease [1–3]. The study of the respiratory microbiome in COPD has revealed a specific bacterial community composition in these patients [4, 5]. However, the relationship between this microbiome and core components of the disease, such as, the severity of the airflow limitation and the type of treatment received remains unclear. In addition, changes in the microbiome have been described in COPD exacerbations [6, 7], but it is not known if differences between patients who suffer two or more exacerbations per year, who are considered frequent exacerbators [8, 9], and non-exacerbators can be detected during clinical stability. Likewise, levels of circulating eosinophils ≥2% in clinically stable patients identifies a subgroup of COPD patients who are prone to recurrent exacerbations and are more responsive to treatment [10–12], but it is not clear if this is associated with a different respiratory microbiome. This work sought to investigate these questions.

## Methods

Methods are detailed in the Additional file 1 and summarized below.

### Study design and ethics

This is a cross-sectional, prospective, uncontrolled, multicentre, observational study. The study protocol was approved by the Ethics Committees of the participating hospitals (IMIM-Hospital del Mar, Hospital Universitari Parc Taulí, Hospital Clinic, Hospital 12 Octubre, Fundación Jimenez Díaz and Hospital Son Espases), and all patients included signed their informed consent.

### Population

Current or former smokers (≥ 10 pack-year) with stable COPD, attending the outpatients’ clinics of five Spanish hospitals between 2014 and 2016 were included in this study. The diagnosis and severity staging of COPD was established in accordance with GOLD criteria [8]. Exclusion criteria were: age less than 40 years; a lifetime diagnosis of asthma, cystic fibrosis, bronchiectasis or cancer; patients receiving long-term treatment with oral corticosteroids or immunosuppressants; any comorbidity limiting cognitive capabilities and ≥ 3 admissions or 1 episode severe enough to require more than 30 days in hospital the previous year. Patients who had been treated with short-term antibiotics and/or corticosteroids at any time during the previous three months were considered unstable and not considered for the study.

### Variables and measurements

Sociodemographic data were recorded by specific questionnaires. Lung function values during stability were obtained from the most recently available forced spirometry with reversibility testing performed according to standard techniques the previous year [13]. Peripheral blood cell counts were obtained at enrolment and used to identify patients with ≥2% circulating eosinophils [14]. Episodes of increased dyspnoea, sputum production and/or purulence during the previous year were identified and considered as exacerbations when treated with antibiotics and/or corticosteroids [15, 16]. Participants were considered as frequent exacerbators (FE) when they reported ≥2 exacerbations the previous year.

### Sample collection, DNA extraction, PCR amplification and 16S sequencing

Spontaneous sputum samples were collected and processed within 60 min on the day of the visit. Sputum quality was assessed according to Murray-Washington criteria [17] and only samples with > 25 leucocytes per field (M-W ≥ 3) were considered for the study. Sputum samples were frozen until processing, which was carried out in a certified BSL2 hood with appropriate laminar flow.16S rRNA gene was amplified following the 16S Metagenomic Sequencing Library Preparation Illumina protocol (Part # 15044223 Rev. A, Illumina, CA, USA). Details are provided in the Additional file 1.

### Sequence analysis

The Quantitative Insights Into Microbial Ecology (QIIME) pipeline 1.9.0 [18] was used for sequence processing to obtain taxonomic information. Further technical details are provided in the Additional file 1.

### Statistical analyses

Details are provided in the Additional file 1. In brief, categorical variables are expressed as absolute and relative frequencies, and continuous variables as means and standard deviations (SD) when the distribution was normal, or as medians and interquartile range (IQR) otherwise. Linear discriminant analysis Effect Size (LEfSe) was used to identify the differentially abundant taxa that explained the differences between the groups of participants. The threshold value of the logarithmic LDA score for discriminative features was 2.0. Bacterial α-diversity was assessed through the Chao1 estimator [19] and the Shannon index [20], calculating both indexes after subsampling with QIIME so as to avoid sequencing effort bias. Principal Coordinates Analysis (PCoA) with Bray-Curtis dissimilarity index [21] was used to study community composition, assessing the statistical significance of the differences in sample groupings through Adonis testing. Interaction between independent variables was assessed through stratification and multivariate analyses with α-diversity as dependent variable. Statistical tests used in the study were two-sided, and a *p* value of 0.05 or less was reported as statistically significant. Statistical analyses were performed using the SPSS statistical software package version 18 (SPSS Inc., Chicago, IL, USA).

## Results

### Patient characteristics

Table 1 summarizes the main demographic and clinical characteristics of the 72 patients included. They were mostly men (88.9%), with a mean age of 68 (SD 7.9) years and FEV1 of 48.7 (SD 20.1)% of reference.

**Table 1:** Demographic and clinical characteristics of the patients

N	All patients	Exacerbations previous year			*p*
		0	1	≥2	
	72	31	18	23	
Age, mean (SD)	68 (7.9)	66 (9)	69 (7)	68 (7)	0.387
Sex (male), *n* (%)	64 (88.9)	27 (87.1)	16 (88.9)	21 (91.3)	0.888
Cumulative smoking (pack-year), median (IQR)	60 (45–80)	60 (44–76)	50 (41–85)	60 (49–97)	0.383
Postbronchodilator FEV1%, median (IQR)	44 (33–60)	52 (42–70)	35 (32–52)	35 (28–49)	0.001
BMI, median (IQR)	27 (24–30)	28 (25–29)	28 (23–30)	26 (23–30)	0.669
Blood eosinophils (× 10^9^/L), median (IQR)	200 (100–270)	200 (130–3009	185 (92–255)	200 (100–300)	0.414
Blood eosinophils (%), median (IQR)	2.4 (1.4–3.4)	2.8 (1.7–3.6)	2 (1.3–2.8)	1.8 (1.1–3.4)	0.156
Blood leucocytes (×10^9^/L), median (IQR)	7845 (6635–9180)	7210 (6520–8940)	7915 (6505–8510)	8110 (7030–10,170)	0.481
Exacerbations last year, median (IQR)	1 (0–2)	0	1	3 (2–4)	
Airflow limitation severity (GOLD), *n* (%)					
GOLD 1	6 (8.3)	5 (16.1)	0 (0)	1 (4.3)	0.013
GOLD 2	19 (26.4)	11 (35.5)	4 (22.2)	4 (17.4)	
GOLD 3	36 (50)	14 (45.2)	12 (66.7)	10 (11.1)	
GOLD 4	11 (15.3)	1 (3.2)	2 (18.2)	8 (34.8)	

### 16S rRNA analysis

At phylum level, 13 different phyla were identified, six of them with median relative abundance (RA) above 0.1% (Additional file 1: Table S1). At genus level, 190 different genera were identified and, after removing the genera present in only one sample, 171 remained for subsequent analyses, 26 of them with RA above 0.1% (Table 2).

**Table 2:** Relative abundance of the genera detected. Only genera appearing in more than one sample and with median relative abundances over 0.1% are shown

Genera	Relative abundance, median (IQR)
*Rothia*	18.65 (9.37–30.33)
*Gemellaceae*_g	7.32 (2.24–13.51)
*Prevotella*	6.87 (2.33–15.05)
*Granulicatella*	4.43 (2.16–6.58)
*Fusobacterium*	2.23 (0.26–3.93)
*Porphyromonas*	1.97 (0.13–8.22)
*Actinomyces*	1.80 (0.58–4.41)
*Streptococcus*	1.92 (1.23–3.44)
*Pseudomonas*	1.39 (0.40–6.36)
*Veillonella*	1.00 (0.50–1.44)
*Atopobium*	0.69 (0.30–1.56)
*Oribacterium*	0.62 (0.15–1.26)
*Leptotrichia*	0.51 (0.09–1.90)
*Lachnospiraceae*_g	0.50 (0.08–1.09)
[*Prevotella*]	0.44 (0.04–1.87)
*Moryella*	0.35 (0.06–0.94)
*Campylobacter*	0.35 (0.10–0.77)
*Capnocytophaga*	0.29 (0.01–0.99)
TM7-3_o_f_g	0.28 (0.04–1.45)
*Megasphaera*	0.25 (0.02–1.17)
*Bulleidia*	0.20 (0.05–0.85)
*Haemophilus*	0.19 (0.05–1.32)
*Selenomonas*	0.18 (0.04–0.62)
*Parvimonas*	0.11 (0.01–0.54)
*Lactobacillus*	0.12 (0.01–1.07)
*Lactobacillales*_Other_Other	0.11 (0.04–0.24)

### Age

Alpha-diversity parameters showed a negative relationship with age (R^2^ = 0.075 *p* = 0.020 and R^2^ = 0.074 *p* = 0.020 respectively), but β-diversity analysis did not show significant differences in relation with this variable (*p* = 0.389).

### Airflow limitation

**Fig. 1:**
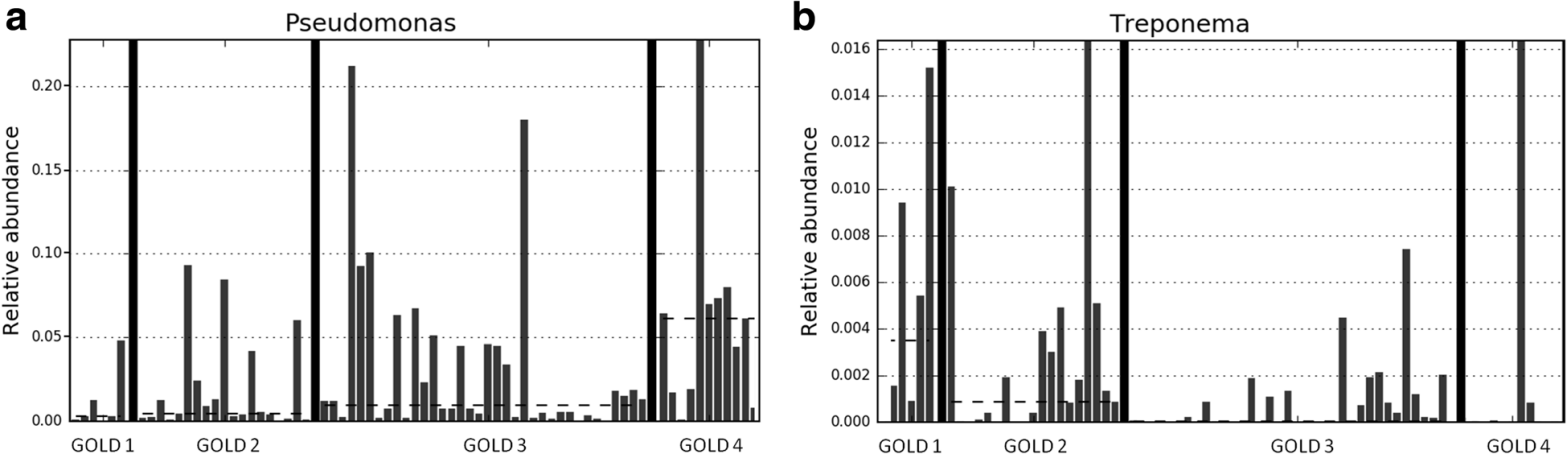
Genera showing significant differences in their relative abundance according to GOLD severity level, with higher figures for *Treponema* in GOLD 1 (**b**) and for *Pseudomonas* in GOLD 4 (**a**) (dotted line = median)

We found a significant progressive increase in the RA of *Pseudomonas* genus and a decrease in the RA of *Treponema* in patients with more severe airflow limitation (Fig. 1). Regarding bacterial diversity, neither α-diversity parameters nor β-diversity analysis showed significant differences between GOLD grades of airflow limitation. Of note, airflow limitation severity was not related to age (*p* = 0.245).

### Pharmacological treatment

Forty-nine COPD patients had not modified their inhaled maintenance treatment during the previous year; thirty-six of them (73.5%) used a combination of LAB/ICS, 9 (18.4%) were treated with LAB as monotherapy and 4 (8.2%) were not receiving COPD treatment. LAB/ICS treatment did not have any effect on either α-diversity (*p* = 0.365) or bacterial community composition in the patients studied (*p* = 0.963), when compared with patients not receiving this treatment. Similarly, the continuous use of LAB as monotherapy was not associated with significant changes in the respiratory microbiome (*p* = 0.854).

### Exacerbation frequency

**Fig. 2:**
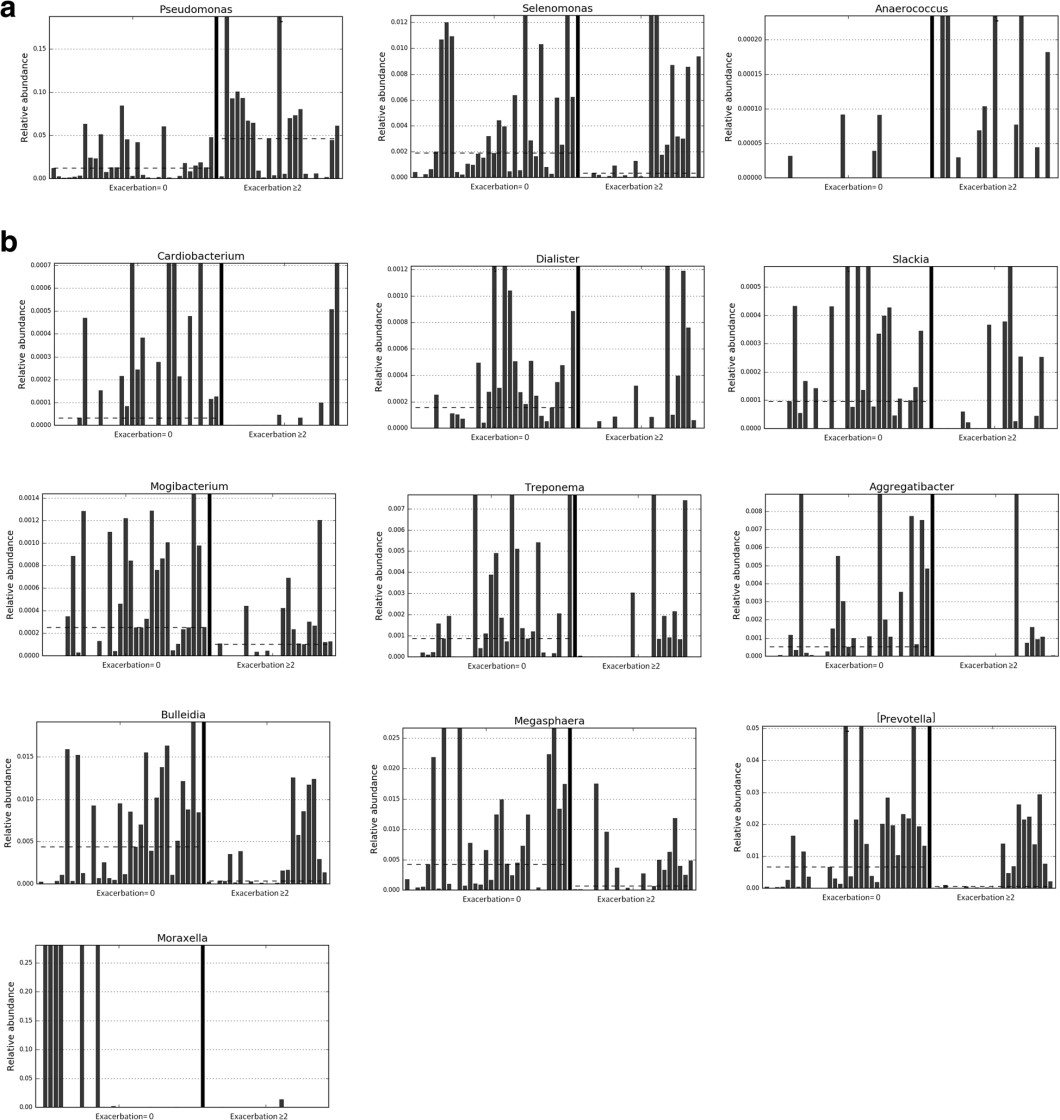
The RA of three genera significantly increased (**a**) and 10 genera decreased (**b**) in patients with ≥2 exacerbations the previous year compared with non-exacerbators (dotted line = median)

In the previous year, 31 patients (43.1%) did not report any acute episodes, 18 (25%) referred only one and 23 suffered two or more (31.9%), and were considered FE. Demographic and clinical characteristics of these three groups only showed statistically significant differences in lung function, with lower values in COPD patients reporting one or more exacerbations the previous year (Table 1) Comparisons between their respiratory microbiomes were made in pairs using patients without exacerbations as the reference. Patients with one exacerbation had significantly lower RA of the phylum TM7 (Additional file 1: Figure S1) and lower RAs of 13 different genera (Additional file 1: Figure S2). However, α-diversity parameters did not show significant differences between the groups, and β-diversity analysis did not demonstrate bacterial communities with a different composition (*p* = 0.081). FE showed a significant decrease in the RA of TM7 and Spirochaetes at phylum level (Additional file 1: Figure S3). At genus level, the RAs of *Pseudomonas*, *Selenomonas* and *Anaerococcus* increased, while 10 different genera decreased (Fig. 2). Alpha-diversity analysis did not show significant differences between groups, but β-diversity analysis demonstrated that the bacterial communities of COPD patients with frequent exacerbations differed significantly (*p* = 0.014).

### Circulating eosinophils

**Fig. 3:**
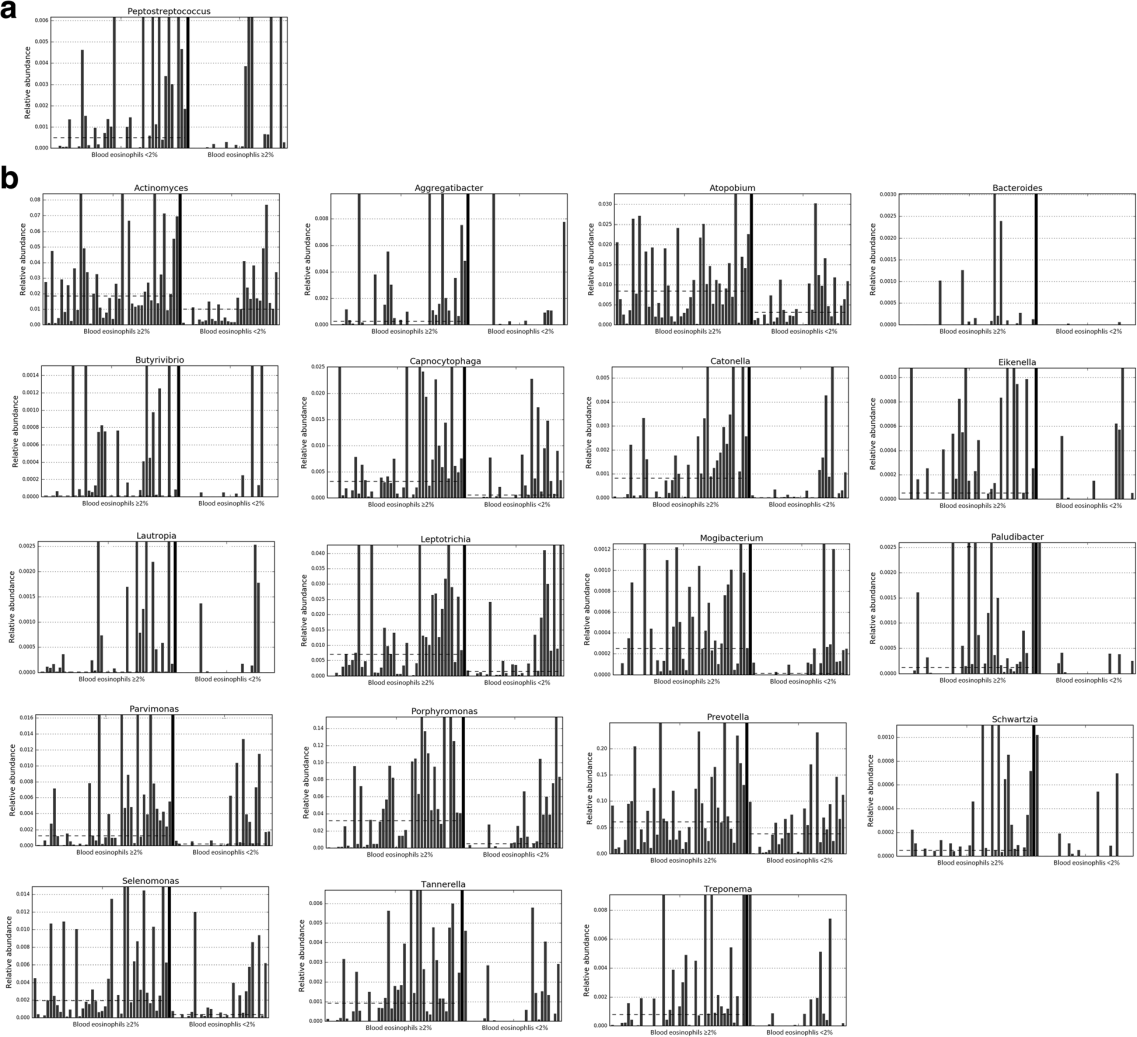
Genera with significantly higher (*n* = 20) (**b**) and lower (**a**) RAs (*n* = 1) in patients with circulating eosinophils ≥2% (dotted line = median)

**Fig. 4:**
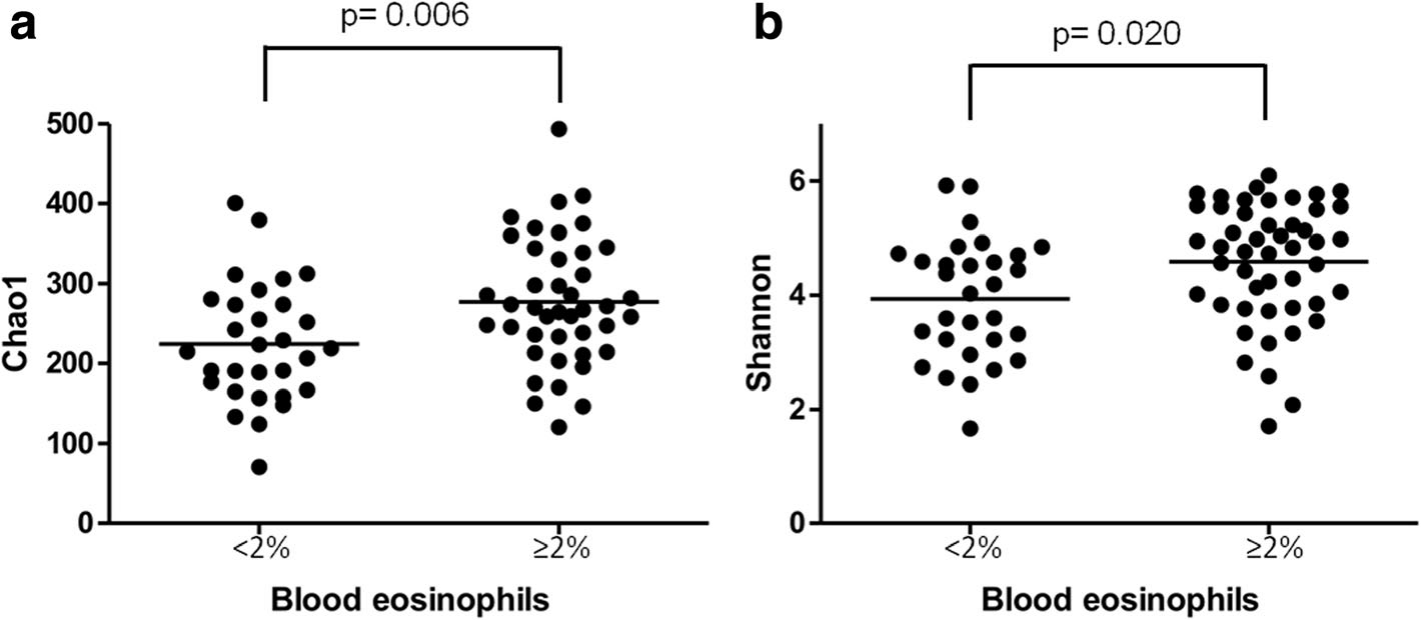
α-diversity parameters, Chao1 (**a**) and Shannon (**b**), in patients classified according to circulating eosinophils ≥2%

Forty-two of the participants (58.3%) had ≥2% blood eosinophils. There were no significant differences in age (*p* = 0.368), sex (*p* = 1.00) and number of exacerbations the previous year (*p* = 0.080) between patients with ≥2% circulating eosinophils or less. The bacterial community in the former had significantly higher RAs of the phyla Bacteroidetes and Spirochaetes (Additional file 1: Figure S4). At genus level, 20 genera showed significantly higher RA and one genus, *Peptostreptococcus,* had lower RA in these patients (Fig. 3). Alpha-diversity was significantly higher in patients with ≥2% circulating eosinophils [Chao1 index: 224.51 (74.88) vs 277.39 (78.92), *p* = 0.006; and Shannon index: 3.94 (1.05) vs 4.54 (1.06), *p* = 0.020] (Fig. 4). Pearson's correlation coefficients were r = 0.282 (*p* = 0.016) for Chao1 and r = 0.231 (*p* = 0.051) for Shannon. β-diversity analysis showed a trend towards different bacterial communities (*p* = 0.072).

Multivariate analyses were performed with α-diversity as dependent variable and eosinophils levels as predictive factor, including age and lung function as covariates. Eosinophils in blood, expressed as percentage, kept a statistically significant relationship with Chao1 in this analysis (*p* = 0.026) and a borderline significance for Shannon (*p* = 0.051), a finding confirming that the bronchial microbiome was related to blood eosinophils independently of the functional limitations suffered by the patient.

To explore potential interactions between the previous history of exacerbations and eosinophils levels, we compared the microbiome in COPD patients with and without circulating eosinophils ≥2% stratified by the frequency of exacerbations. We found that the significant differences in the microbial composition related to patients with eosinophils ≥2% were maintained in the subsample of patients with no exacerbations or only one episode (*p* = 0.033), but this effect disappeared in FE (*p* = 0.995).

## Discussion

The main findings of this study were that the diversity and composition of the respiratory microbiome in clinically stable COPD patients change in relation to age, the severity of airflow limitation, exacerbation frequency and eosinophils in peripheral blood.

In our study, older age was significantly associated with a loss of diversity, which has been also found in the gut microbiome [22]. Besides, in patients with severe asthma, an inverse correlation between α-diversity and age has been also reported [23]. A previous work has shown less microbial diversity of the respiratory microbiome in younger COPD patients using bronchoalveolar lavage [24], but this sample targets the peripheral airway of the lung and it is not representative of the bronchial tree mainly sampled by sputum [25].

We found that patients with more severe airflow limitation had a significant decrease in the RA of *Treponema* and a progressive increase in the RA of *Pseudomonas*. These results suggest that severity-related changes in the respiratory microbiome are based on a decrease in specific genera, which are partially substituted by *Pseudomonas*. This change may be partly related to recurrent antibiotic exposure in previous years, considering the antibiotic sensitivity of the microorganisms part of *Treponema* genus. Previous cross-sectional studies evaluating the relation between bacterial diversity and more severe airway limitation have mostly showed a decline in advanced stages [26–28], associated with changes in the RAs of specific genera such as *Haemophilus* [28, 29]. These partly discordant results may be due to patient selection, considering that most of the previous studies have focused on a restricted number of patients with moderate or severe disease [26, 27] or an overrepresentation of patients with moderate disease [28] whereas we studied a wider range of disease severity (GOLD 1–4). Our results, therefore, support a significant role for *Pseudomonas* as the severity of the disease increases to higher lung function impairment.

We also found that the respiratory microbiome was significantly different in FE. Previous studies have investigated the characteristics of the respiratory microbiome during exacerbations [7, 30], and recently, like we do in this study, Mayhew and cols. [28] reported specific characteristics in the bronchial microbiome recovered from FE patients during clinical stability. Both studies show that FE have a different respiratory microbiome during clinical stability, suggesting that the microbial changes during exacerbations in FE may be a mixture of the dysbiosis found in stability and specific exacerbation-related perturbations of the lung bacteria community composition [28, 31].

Circulating eosinophils ≥2% were associated with higher microbial diversity in the population studied. Patients with ≥2% blood eosinophils have been reported to have more frequent exacerbations and a better response to ICS preventive therapy [32] Previous studies have demonstrated a different bronchial microbiome in eosinophilic COPD exacerbations [7, 31], which seems related to Th2 inflammation in both COPD and asthma [33]. In our study we observed that in patients with ≥2% blood eosinophils higher microbial diversity is already present in stability, with an increase in the RA of 20 genera. Similar results have been reported in stable asthmatic patients, who showed a good correlation between the percentage of eosinophils in bronchoalveolar lavage and bacterial diversity [34]. Higher bacterial diversity may have a protective role in patients with ≥2% blood eosinophils avoiding the presence of pathogenic bacteria such as *Haemophilus influenzae* and *Streptococcus pneumonia* which has been reported to be overrepresented in patients with eosinophils counts below 2% treated with ICS [12]. Yet, when we stratified the COPD patients included according to both the level of circulating eosinophils and the frequency of exacerbations, we observed that the differences related to blood eosinophils disappeared in FE, likely highlighting a higher impact of frequent exacerbations on the respiratory microbiome in these patients.

This study has some potential limitations. First, we do not have a wide representation of the respiratory microbiome, which has been shown to be heterogeneous throughout the airway, because only sputum samples were analysed. Second, although the patients included had not taken antibiotics three months before their inclusion, we lack information on previous antibiotic treatments, which may have had an effect on their microbial communities. Finally, we analysed only bacterial communities, fungi and virus may also have an effect on these patients, either directly or through interactions with other microorganisms and the host.

## Conclusions

This study shows that the respiratory microbiome in clinically stable COPD patients changes in relation to age, severity of airflow limitation, history of previous exacerbations and level of circulating eosinophils. These factors need to be considered when interpreting respiratory microbiome changes in patients with COPD.

## Additional file

Additional file 1: **Table S1.** Relative abundances of the phyla detected. **Figure S1.** The TM7 phylum had significantly lower relative abundance in patients with one exacerbation than patients without exacerbations the previous year (dotted line = median). **Figure S2.** Thirteen genera with significantly lower relative abundances in COPD patients with one exacerbation the previous year compared to non-exacerbators. **Figure S3.** A significant reduction in the RA of phyla TM7 and Spirochaetes in patients with ≥2 exacerbations the previous year, using patients without exacerbations as the reference (dotted line = median). **Figure S4.** Phyla with significantly higher relative abundances in COPD patients showing circulating eosinophils ≥2%. (DOCX 725 kb)

## Data Availability

Bacterial 16SrRNA datasets from this study are accessible in the European Nucleotide Archive under the study PRJEB26773 with the sample numbers ERS2486515–609.

## Abbreviations

COPD: Chronic obstructive pulmonary disease
DNA: Deoxyribonucleic acid
FE: Frequent exacerbators
FEV1: Forced expiratory volume the first second
GOLD: Global Initiative for Chronic Obstructive Lung Disease
ICS: Inhaled corticosteroids
IQR: Interquartile range
LAB: long-acting bronchodilator
LDA: Linear discriminant analysis
LEfSe: Linear discriminant analysis Effect Size
PCoA: Principal Coordinates Analysis
PCR: Polymerase chain reaction
QIIME: Quantitative Insights Into Microbial Ecology
RA: Relative abundance
rRNA: Ribosomal ribonucleic acid
SD: Standard deviations
vs: Versus
