# Supplementary material for: Relationship between the respiratory microbiome and the severity of airflow limitation, history of exacerbations and circulating eosinophils in COPD patients
Source: BMC Pulm Med. 2019 Jun 24;19:112. doi: 10.1186/s12890-019-0867-x (PMC6591812; doi:10.1186/s12890-019-0867-x)
Supplement: Supplementary file 1 — Table S1. Relative abundances of the phyla detected. Figure S1. The TM7 phylum had significantly lower relative abundance in patients with one exacerbation than patients without exacerbations the previous year (dotted line = median). Figure S2. Thirteen genera with significantly lower relative abundances in COPD patients with one exacerbation the previous year compared to non-exacerbators. Figure S3. A significant reduction in the RA of phyla TM7 and Spirochaetes in patients with ≥2 exacerbations the previous year, using patients without exacerbations as the reference (dotted line = median). Figure S4. Phyla with significantly higher relative abundances in COPD patients showing circulating eosinophils ≥2%. (DOCX 725 kb) [file 12890_2019_867_MOESM1_ESM.docx]

**METHODS**

**Sample collection and DNA extraction**

Sputum samples were thawed and incubated with four volumes of Sputasol (Oxoid, Hampshire, UK) at room temperature for 15 minutes. Then, the same volume of phosphate-buffered saline (PBS) was added, mixed and centrifuged at 16000 g for 10 minutes. Sputum pellets were mixed with 0.6 mL of a lysis buffer containing 83 ug/mL lysozyme (Sigma-Aldrich Corp., St. Louis, MO, USA), 20 U/mL lysostaphin (Sigma-Aldrich) and 250 U/mL mutanolysin (Sigma-Aldrich), and incubated for two hours at 37°C [1]. Then, samples were transferred to a DNA Dry bead tube (Lysing Matrix A, MP Biomedicals, Santa Ana, CA, USA) and shaken at 5000 rpm for 30 seconds in a Precellys Minilys homogenizer (Bertin Technologies, Rockville, Washington, DC, USA). Proteinase K was added to the samples and, after incubation for one hour at 60ºC, they were homogenized again. DNA was purified with DNeasy Blood & Tissue Kit (Qiagen, Helden, Germany) following the manufacturer’s instructions and quantified with Qubit Fluorometer (Life Technologies, Thermo Fisher Scientific, Waltham, MA, USA).

**PCR amplification and sequencing of 16S rRNA gene**

16S was amplified following the 16S Metagenomic Sequencing Library Preparation Illumina protocol (Part # 15044223 Rev. A, Illumina, CA, USA). The gene‐specific sequences used in this protocol target the 16S V3 and V4 region. Illumina adapter overhang nucleotide sequences were added to gene‐specific sequences, and primers were selected following Klindworth and cols. [2]. Using the standard IUPAC nucleotide nomenclature, the full length primer sequences used to follow the protocol targeting this region were: 16S Forward primer = 5'- tcgtcggcagcgtcagatgtgtataagagacagcctacgggnggcwgcag-3’ and reverse primer= 5'- gtctcgtgggctcggagatgtgtataagagacaggactachvgggtatctaatcc-3’.

Microbial Genomic DNA (5 ng/μl in 10 mM Tris pH 8.5) was used to initiate the protocol. PCR conditions were 5 min of initial denaturation at 94 ºC followed by 25 cycles of denaturation (30 s at 94 ºC), annealing (30 s at 52 ºC) and elongation (1 min at 72 ºC). After amplification, the products were visualized in 2% agarose gels. Four sputum-processing controls and six extraction controls were PCR amplified in parallel with the samples, and, although no bands were detected in the gel electrophoresis, they were sequenced together with the samples. After 16S amplification, the multiplexing step was performed using Nextera XT Index Kit (FC-131-1096, Illumina). One microliter of the PCR product was run on a Bioanalyzer DNA 1000 chip (Agilent, CA, USA) to verify the expected size of ~550 bp. After size verification, the libraries were sequenced using a 2x300 bp paired-end run (MiSeq Reagent kit v3 MS-102-3001, Illumina), on a MiSeq Sequencer according to the manufacturer’s instructions (Illumina).

Quality assessment was performed using the PRINSEQ-lite program [3] with the following parameters: min_length: 50,trim_qual_right: 20, trim_qual_type: mean, trim_qual_window: 20. R1 and R2 from Illumina sequencing were joined using fastq-join from ea-tools suite [4].

**Sequence analysis**

The Quantitative Insights Into Microbial Ecology (QIIME) pipeline 1.9.0 [5] was used for sequence processing to obtain taxonomic information using the Greengenes 13_8 sequence database as reference and the RDP classifier 2.2. The open reference operational taxonomic unit (OTU) picking method was used with UCLUST and PyNAST version 1.2.2 as alignment method. Chimeric sequences were detected in QIIME with ChimeraSlayer and were removed from the OTU table and from the phylogenetic tree to perform downstream analyses.

In order to assess the influence of the reagent contamination in our samples, we sequenced four sputum-processing controls and six extraction controls. We obtained a mean of 11,173.89 (SD 11,548.71) sequences in these controls, which were processed in QIIME as were the samples. We detected 20 phyla and 133 genera in the controls, 78 of them with a relative abundance >1% in at least one sample.

Following Bittinger and cols. [6], Fisher exact test was used to compare the overall frequency of occurrence of each genus between samples and controls. Genera showing relative abundances (RAs) in controls exceeding those in samples were considered as potential contaminants and were removed from the subsequent analyses, and common contaminant genera were also checked in the samples and eliminated when present [7]. After removing all the contaminant OTUs from the final OTU table, downstream analyses were performed to determine alpha and beta-diversity.

**Statistical analyses**

Linear discriminant analysis Effect Size (LEfSe) was used to identify the differentially abundant taxa.. This algorithm uses Linear Discriminant Analysis (LDA) to estimate the effect size of each differentially abundant feature [8].

First, the bronchial microbiome of the participants was related to their age, severity, according to GOLD levels, and treatment. Second, exacerbation frequency the previous year was used to classify the COPD patients as non-exacerbators, exacerbators (patients with one moderate/severe episode) and frequent exacerbators (FE) (≥2 episodes) and their microbiomes were compared in pairs. Third, participants categorized according to blood eosinophils ≥2% were identified and their microbiome compared with the other participants. Interactions between exacerbation frequency and levels of eosinophils ≥2 were also assessed. Finally, interactions between exacerbation frequency, levels of eosinophils and the measured covariates were assessed through multivariate analyses with α-diversity as dependent variable.

1. Yuan S, Cohen DB, Ravel J, Abdo Z, Forney LJ. Evaluation of methods for the extraction and purification of DNA from the human microbiome. PloS One. 2012;7:e33865.

2. Klindworth A, Pruesse E, Schweer T, Peplies J, Quast C, Horn M, et al. Evaluation of general 16S ribosomal RNA gene PCR primers for classical and next-generation sequencing-based diversity studies. Nucleic Acids Res. 2013;41:e1.

3. Schmieder R, Edwards R. Quality control and preprocessing of metagenomic datasets. Bioinforma Oxf Engl. 2011;27:863–4.

4. Erik Aronesty. ea-utils : “Command-line tools for processing biological sequencing data”; Httpcodegooglecompea-Utils. 2011.

5. Caporaso JG, Kuczynski J, Stombaugh J, Bittinger K, Bushman FD, Costello EK, et al. QIIME allows analysis of high-throughput community sequencing data. Nat Methods. 2010;7:335–6.

6. Bittinger K, Charlson ES, Loy E, Shirley DJ, Haas AR, Laughlin A, et al. Improved characterization of medically relevant fungi in the human respiratory tract using next-generation sequencing. Genome Biol. 2014;15:487.

7. Salter SJ, Cox MJ, Turek EM, Calus ST, Cookson WO, Moffatt MF, et al. Reagent and laboratory contamination can critically impact sequence-based microbiome analyses. BMC Biol. 2014;12:87.

8. Segata N, Izard J, Waldron L, Gevers D, Miropolsky L, Garrett WS, et al. Metagenomic biomarker discovery and explanation. Genome Biol. 2011;12:R60.

TABLES

Table S1, supplementary material**.** Relative abundances of the phyla detected. Only phyla appearing in more than one sample and with median relative abundances above 0.1% are shown.

| **Phylum** | **Relative abundance, median (IQR)** |
| --- | --- |
| Firmicutes | 29.12 (19.60-36.95) |
| Actinobacteria | 24.84 (16.05-37.88) |
| Bacteroidetes | 14.94 (5.20-26.12) |
| Proteobacteria | 7.32 (3.30-17.43) |
| Fusobacteria | 3.46 (0.54-6.49) |
| TM7 | 0.78 (0.06-2.06) |

**FIGURES**





Figure S1, supplementary material. The TM7 phylum had significantly lower relative abundance in patients with one exacerbation than patients without exacerbations the previous year (dotted line=median).





Figure S2, supplementary material. Thirteen genera with significantly lower relative abundances in COPD patients with one exacerbation the previous year compared to non-exacerbators.





Figure S3, supplementary material. A significant reduction in the RA of phyla TM7 and Spirochaetes in patients with ≥2 exacerbations the previous year, using patients without exacerbations as the reference (dotted line=median).





Figure S4. supplementary material. Phyla with significantly higher relative abundances in COPD patients showing circulating eosinophils ≥2%.
